# Psychological Status Associated With Low Quality of Life in School-Age Children With Neurodevelopmental Disorders During COVID-19 Stay-At-Home Period

**DOI:** 10.3389/fpsyt.2021.676493

**Published:** 2021-10-18

**Authors:** Riyo Ueda, Takashi Okada, Yosuke Kita, Yuri Ozawa, Hisami Inoue, Mutsuki Shioda, Yoshimi Kono, Chika Kono, Yukiko Nakamura, Kaoru Amemiya, Ai Ito, Nobuko Sugiura, Yuichiro Matsuoka, Chinami Kaiga, Masaya Kubota, Hiroshi Ozawa

**Affiliations:** ^1^Department of Developmental Disorders, National Institute of Mental Health, National Center of Neurology and Psychiatry, Kodaira, Japan; ^2^Department of Child Neurology, Shimada Ryoiku Center Hachioji, Hachioji, Japan; ^3^Mori Arinori Center for Higher Education and Global Mobility, Hitotsubashi University, Kunitachi, Japan; ^4^Cognitive Brain Research Unit (CBRU), Faculty of Medicine, University of Helsinki, Helsinki, Finland

**Keywords:** COVID-19, children, neurodevelopmental disorders, quality of life, depression

## Abstract

**Background:** This study seeks to ascertain how the COVID-19 stay-at-home period has affected the quality of life (QOL) of children with neurodevelopmental disorders (NDDs) who had experienced sleep schedules alteration and clarify what psychological status predicted low QOL in children with and without altered sleep patterns.

**Materials and Methods:** Study participants were 86 children between 8 and 17 years of age (mean age, 11.7 years; 70 boys, 16 girls; mean intellectual quotient, 83.6). QOL was evaluated using the self-assessment KINDL^R^. Participants answered questions regarding depression and anxiety on a visual analog scale (VAS) for temporary mood. Their parents answered questionnaires regarding their maladaptive behaviors and differences in sleep patterns before and during the COVID-19 pandemic. The student's *t*-test was performed to examine the presence or absence of sleep changes in the children, which affected QOL, temporary mood, and maladaptive behaviors. Multiple or simple linear regression analyses were also performed to identify the psychogenic factors that significantly affected decreased QOL for each group with and without changes in sleep schedule.

**Results:** During the COVID-19 stay-at-home period, 46.5% of participants experienced changes in sleep patterns. These changes were associated with decreased QOL as well as internalized symptoms. The decreased QOL of children with sleep patterns changed was predicted by a high level of depression. In addition, low QOL in children with unchanged sleep patterns was predicted by a high level of depression and low current mood status.

**Conclusions:** Almost half of the participants experienced a poor sleep schedule during the stay-at-home period. These alterations in sleep patterns were associated with a low QOL. The QOL of children with a stable life schedule was affected not only by depressive tendencies but also temporary moods. Therefore, they need to live a fulfilling life to maintain their QOL. However, the QOL of children with poor sleep patterns was affected only by depressive tendencies. Hence, clinicians need to ensure that children with NDDs are well-diagnosed with depression and treated for sleep problems.

## Introduction

The 2019 coronavirus disease (COVID-19) pandemic has profoundly altered the way people live and work worldwide. In particular, the suspension of in-person education, extracurriculars, social activities, and routine healthcare for children severely threatens their physical and mental well-being.

In Japan, an emergency declaration was issued by the Prime Minister on April 7, 2020. Citizens were mandated to stay at home and refrain from outdoor activities until May 25, 2020. School-age children had been sent home even earlier, on March 2, 2020. Similar to caregivers around the world, Japanese parents too faced questions about how to best support their children under these conditions (1, 2).

Neurodevelopmental disorders (NDDs) are a group of conditions that produce developmental impairment in personal, social, and academic functioning from the developmental period, including genetic syndromes, metabolic diseases, cerebral palsy, psychomotor delay, etc. (3). It has been warned since the beginning of the COVID-19 pandemic that children with NDDs are more likely to experience mental and physical difficulties during a disaster in comparison with typically developing children (TDC) because of their inability to adapt to unpredictable changes around them and alterations to their routines (1, 4, 5). In the Australian study, the lifestyle habits of children with NDDs worsened, especially in terms of spending more time watching TV and digital media, getting less exercise, and having a poor diet (6). Furthermore, it was revealed in several studies that both internalizing and externalizing symptoms in children with NDDs and their comorbid mental health symptoms were worse than before the COVID-19 outbreak (1, 6). In addition, one of the severe problems for caregivers was the increased psychological and social burden of parenting children (7–10). The suspension of daily rehabilitation services and the lack of alternative recreational opportunities leave these caregivers alone caring for their children, with increased childcare burden and stressors, such as lack of access to needed therapies, medical supplies, and nursing care (7–10). The concerns for their child were significantly associated with the caregivers' stress, depressive and anxious symptoms (9). Namely, in recent years, the correlation between the social status of children with NDDs and the psychological status of their parents as well as the worsening of children's psychological behaviors during the COVID-19 pandemic has been revealed, emphasizing the importance of appropriate assessment and alternative intervention for children with NDDs as one of the global public health priorities (1, 6–9, 11).

Among the many problems of children with NDDs during the COVID-19 pandemic, deterioration of sleep schedules was one of the most significant problems. Children with NDDs are prone to sleep problems, even under normal circumstances. Sleep dysfunction for children with NDDs was a common and underlying problem associated with multiple factors, including biological and genetic abnormalities (12–14). Previous studies have shown that sleep problems affect the mental problems of children with the attention-deficit hyperactive disorder (ADHD) and autism spectrum disorder (ASD) (15–17). Some neurological and psychological experts predicted sleep problems in children with NDDs, especially during the COVID-19 pandemic (4). Therefore, it is significant to identify changes in sleep patterns during the COVID-19 stay-at-home period and related factors.

Furthermore, improving the subjective indicator of children's well-being is one of the most important goals in terms of supporting children. Quality of life (QOL) describes an individual's subjective perception of their position in life, as evidenced by their physical, psychological, and social functioning (18).

In a previous study, we asked parents to respond to the questionnaire survey on changes in children's QOL and lifestyle during the COVID-19 stay-at-home period. We found that changes in children's sleep schedules were associated with reduced QOL, and decreased maladaptive behavior with a maintained QOL (19). Thus, our former study indicated the importance of adjusting the sleep schedule to maintain the QOL of children. However, many previous studies have shown that the results of parental proxy- and children's self-reports do not always match (20–23). Therefore, it is prudent to analyze the results of the children's assessment of their mental state and QOL and to clarify their detailed characteristics.

The purpose of this study was to determine, using self-assessments, how the QOL of school-age children with NDDs during the COVID-19 pandemic has been affected by changes in the sleep schedules and the psychological status that predicted low QOL in children with and without altered sleep patterns. Clarifying the relationship between children's sleep problems and QOL can help reveal focus areas for child care and its support.

## Materials and Methods

### Participant Characteristics

We recruited 86 children who were patients at the Shimada Ryoiku Center Hachioji in May 2020. The center is a regional core outpatient clinic where children receive medical examinations, rehabilitation, and psychotherapy. Hachioji is located in the western suburbs of Tokyo. It is a commuter town with a population of 580,000 (population density 3,093/km^2^). Nineteen persons were infected with COVID-19 in Hachioji City (a 6.8% positivity rate in polymerase chain reaction examination for COVID-19) in May 2020.

The inclusion criteria for participants were children with NDDs, including ADHD, ASD, specific learning disorders (SLD), tic disorders, or neurodevelopmental disorders classified by DSM-5. All diagnoses were reviewed by two board-certified pediatricians, including at least one board-certified pediatric neurologist. The children were between 8 and 17 years of age, referencing the target age of the questionnaires. The exclusion criterion was children with moderate or profound intellectual disabilities through prior testing full scale intellectual quotient (FSIQ) score from the Wechsler Intelligence Scale for Children, 3rd edition or 4th edition.

In addition, the center's dedicated staff explained the study to all participants who met the above criteria while maintaining social distance in a well-ventilated large room. All parents agreed to the participation and provided written informed consent, as did the children. We collected the questionnaire from all the children who provided their informed consent.

No one refused to participate. The studies involving human participants were reviewed and approved by the Institutional Review Board of the Shimada Ryoiku Center Hachioji (Shimahachi-2001). The participants and their parents provided their written informed consent to participate in this study.

### Measures

The children were asked to self-administer the following questionnaires to assess their clinical status: Kidd-KINDL^R^ (8–13 years) or Kiddo-KINDL ^R^ (14–17 years) (24, 25), the Depression Self-Rating Scale for Children (DSRS-C) (26), the Spence Children's Anxiety Scale (SCAS) (26, 27), and a visual analog scale (VAS) for temporary mood status in terms of percentages of the best imaginable state (28, 29). The parents assessed the maladaptive behaviors of their children using the Child Behavior Checklist (CBCL) (30). They also answered whether their children were going to bed or waking up later during the COVID-19 stay-at-home period than before.

The KINDL^R^ items were rated on a five-point Likert scale, and the mean scores for each subscale and total items were calculated and converted to a 0–100 scale. The average values in the Kiddo-KINDL^R^ of four subscales (physical well-being, emotional well-being, self-esteem, and family), excluding social contact and school sub-scales, were calculated to evaluate the children's QOL. Higher KINDL^R^ scores indicate a better QOL.

Furthermore, VAS includes a graph scale on a horizontal line with endpoint 0 (the worst mood status imaginable, the picture of a crying face), mid-point 50 (intermediate mood status, the picture of a neutral face), and opposite endpoint 100 (the best mood status imaginable, the picture of a smiling face). The face scale was added to VAS to increase non-verbal explanations for children with different verbal abilities (31). After being instructed on VAS by a pediatrician, participants were asked to mark across the line on a point from (inclusive) 0 to 100, which best describes their current mood state. Higher VAS scores were associated with better mood scores and vice versa, while scores of the other questionnaires showed better symptoms at lower scores.

### Statistical Analysis

Statistical analysis was conducted using the JMP software, version 9.0.3 (SAS Institute Inc., Cary, NC, USA). Students *t*-test was performed to examine the presence or absence of sleep changes in the children, thereby affecting their QOL (KINDL^R^), VAS, FSIQ, externalized index, and internalized index of the CBCL. Pearson's χ^2^-test was used to investigate the proportional differences of the children with sleep changes between presence or absence of ADHD, ASD, and SLD, respectively. We applied a stepwise multiple or simple linear regression analysis to QOL with selective pairs to identify the psychogenic factors for each group, with and without a sleep schedule change. We began with a model that included all of the psychogenic available explanatory variables: SCAS score, DSCR-C score, externalized index and internalized index of the CBCL, and VAS score; we subsequently dropped insignificant variables based on the Akaike information criterion and the Bayesian information criterion. Significance was set at *P* < 0.05.

## Results

### Participants' Background

Table 1 shows the demographic parameters of the study participants. We surveyed their background and sleep schedules and assessed their QOL and mental health. There were 46 (53.5%) children with unchanged sleep schedules and 40 (46.5%) with changed sleep schedules (26 children with later bedtimes, 13 children with later bedtimes and later waking times, and one child with a later waking time than before the COVID-19 pandemic) (Table 1). None of the children took hypnotics and/or were newly diagnosed with a sleep disorder during the COVID-19 stay-at-home period.

**Table 1:** Clinical background of children (*n* = 86).

	**Total**
Male: Female	70:16
Age M ± SD	11.7 (2.2)
WISC/WAIS FSIQ M ± SD	83.6 (16.2)
ADHD *N*, (%)	50 (58.1)
ASD *N*, (%)	43 (50.0)
SLD *N*, (%)	5 (5.8)
Alteration of sleep schedules *N*, (%)	40 (46.5)
Bedtime later than before *N*, (%)	26 (30.2)
Arise time later than before *N*, (%)	1 (1.2)
Both bedtime and arise time later than before *N*, (%)	13 (15.1)

*(%) data indicate the proportion of each characteristic in every group*.

*N, number; M, mean; SD, standard deviation; FSIQ, full-scale intellectual quotient; ADHD, attention-deficit hyperactivity disorder; ASD, autism spectrum disorder; SLD, specific learning disorder*.

Table 2 shows the results of the questionnaires. Among participants, 36 children (41.9%) scored above the CBCL internalized index cut-off of ≥70 points for the clinical range, and 26 children (30.2%) scored above the CBCL externalizing score cut-off of ≥70 points for the clinical range. Of the participants, 19 (22.1%) scored above the DSCR-C cut-off of ≥16 points, and another 19 (22.1%) scored above the SCAS cut-off of ≥42 points (27). The median QOL score for children was 71.2 (out of 100.0) on the KINDL^R^ questionnaire. The median score of temporary mood for children was 55.8 out of 100.0 points on the VAS scale.

**Table 2:** Results of questionnaires.

**Questionnaire: children**	**Median, range**	***N*** **(≥cut-off) (%)**	**Cut-off**
Total scores of KINDL^R^	71.3, 22.5–97.5		—
Physical health	80, 20–100		—
Emotional well-being	80, 25–100		—
Self-esteem	60, 20–100		—
Family	75, 25–100		—
CBCL, internalized index	66, 41–93	36 (41.9)	≥70
CBCL, externalized index	67, 39–91	26 (30.2)	≥70
DSRS-C	11, 0–26	19 (22.1)	≥16
SCAS	27.5, 0–71	19 (22.1)	≥42
VAS for mood	55.8, 0–100		—

*N, number; CBCL, Child Behavior Checklist; DSRS-C, Depression Self-Rating Scale for Children; SCAS, Spence Children's Anxiety Scale; VAS, visual analog scale*.

### Sleep Schedules Alteration of Children During COVID-19 Stay-At-Home

Table 3 shows the relationship between changes in sleep schedules and QOL in children during the COVID-19 stay-at-home period (deterioration = 40; unchanged = 46). Changes in sleep schedules were associated with decreased QOL and were also associated with the internalized index of CBCL. Changed sleep schedules were not associated with the externalized index and FSIQ. There was no relationship between sleep schedules and NDDs (ADHD, ASD, and SLD).

**Table 3:** Relationship between changes in sleep schedules and QOL changes in children during the COVID-19 stay-at-home period.

	**Deterioration of sleep schedules**	**Unchanged sleep schedules**	***t-*** **value/χ^2^-value**	***p-*** **value**
Kiddo-KINDL M; SD	66.8 (14.4)	75.9 (11.9)	3.220^a^	0.002*
VAS score M; SD	57.4 (25.4)	64.3 (22.9)	1.342^a^	0.183
CBCL; internal M; SD	69.4 (11.8)	63.5 (9.9)	2.547^a^	0.013*
CBCL; external M; SD	68.1 (10.7)	64.2 (10.9)	1.683^a^	0.096
FSIQ M; SD	86.6 (12.6)	81.0 (18.5)	1.603^a^	0.113
ADHD (%)	26 (52.0)	24 (48.0)	1.446^b^	0.229
ASD (%)	22 (51.2)	21 (48.8)	0.748^b^	0.387
LD (%)	2 (40)	3 (60)	0.388^b^	0.533

*(%) data indicate the proportion of each characteristic in every group*.

* *p < 0.05*.

*QOL, quality of life; N, number; M, mean; SD, standard deviation; FSIQ, full scale intellectual quotient; ADHD, attention-deficit hyperactivity disorder; ASD, autism spectrum disorder; SLD, specific learning disorder; VAS, visual analog scale*.

a *t-value*.

b *χ^2^-value are depicted*.

### Psychogenic Factors Predicting Lower QOL

Table 4 shows the psychological factors that significantly predicted the QOL of children. Stepwise multiple regression analysis for children with changed sleep schedules showed that higher DSCR-C scores were associated with lower KINDL^R^ scores. In children with unchanged sleep schedules, higher DSCR-C scores, and lower VAS scores were associated with lower KINDL^R^ scores.

**Table 4:** Results of multiple or simple regression analysis for KINDL^R^ score predicting lower QOL.

**Parameter**	**Unchanged sleep schedules (*****n*** **= 46)**		**Changed sleep schedules (*****n*** **= 40)**	
	***t*** **-value**	**β coefficient**	***t*** **-value**	**β coefficient**
Intercept	20.51***	–	36.17***	
DSRS-C	7.99***	−0.697	10.98***	−0.872
VAS for mood	3.75***	0.327	N/A	N/A
*F*-value	47.1***		120.6***	
*R* ^2^	0.69***		0.76***	

*QOL, quality of life; DSRS-C, Depression Self-Rating Scale for Children; VAS, visual analog scale*.

*** *p < 0.001*.

## Discussion

To our knowledge, this was the first study to reveal the relationship between changed sleep patterns in children with NDDs and their QOL during the COVID-19 stay-at-home period, based on the results of self-assessment by children. Changed sleep patterns were associated with decreased QOL and internalized symptoms of children with NDDs. The decreased QOL of children with changed sleep patterns was predicted by high levels of depression. In addition, low QOL in children with unchanged sleep patterns was predicted by high levels of depression and low current mood status.

### Factors Related to Changed Sleep Patterns

Results indicate that changed sleep patterns were associated with decreased QOL in our previous study. In previous studies, children with ADHD and ASD were prone to sleep problems, affecting their QOL, even when they were not facing a crisis (15–17). A Turkish study during the COVID-19 pandemic indicated that severe sleep disorders led to highly increased ASD symptoms (32). In our previous study, QOL of children in parent proxy-reports also decreased with changes in sleep during the COVID-19 stay-at-home period (19). Despite a weak correlation of the same domain in both reports in a previous study (20), the similarity of the results in self-report and proxy-report was clarified in this study. Conversely, there was no relationship between VAS scores and changes in sleep patterns in this study. Since the sleep schedule is not related to temporary mood status during daytime (VAS), it is recommended that parents and clinical practitioners monitor children's sleep conditions.

In addition, changed sleep patterns were associated with maladaptive behavior in children during the COVID-19 pandemic. In previous studies, internalizing symptoms were associated with problematic sleep behaviors in children with comorbid ASD or ADHD, even in non-emergency situations (33, 34). During the COVID-19 stay-at-home period in Italy, it was revealed that ASD children had more intense and frequent disruptive behavior, although there was no mention of the relationship between sleep problems and maladaptive behavior (1). A similar relationship was also clarified during the stay-at-home periods in the present study.

In summary, the relationship between sleep schedule change and low QOL, and between sleep schedule change and internalized symptoms in children with NDDs, during the COVID-19 stay-at-home period tended to be the same as before the COVID-19 pandemic.

### Relationship Between QOL and Psychogenic Status

The decreased QOL of children with changed sleep patterns was predicted by high levels of depression. Furthermore, low QOL in children with unchanged sleep patterns was predicted by high levels of depression and low current mood status. As in previous studies, the prediction of low QOL was associated with increased depressive symptoms, regardless of sleep problems (35–37). Furthermore, in children with unchanged sleep patterns, worse temporary mood status was also a predictor of lower QOL. A positive relationship between temporary mood status and QOL in children having unchanged sleep patterns is affected by sleep invoked sufficient emotional adjustment (38).

In summary, the QOL of children with a stable life schedule was affected not only by depressive tendencies but also temporary moods. Therefore, they need to live a fulfilling life to maintain their QOL. However, the QOL of children with poor sleep patterns was affected only by depressive tendencies. Hence, it is important for clinicians that children with NDDs are correctly diagnosed with depression and treated for sleep problems.

In addition, the telerehabilitation (online rehabilitation) and online medical service for children with NDDs were constructed in several countries during the prolonged COVID-19 pandemic to continue care and adequate support to children and their families (39–41) and to ensure that the human rights of children with NDDs are protected, even during the emergency (42). It has been paying attention because of its ability to at least partially reduce the risk of hopelessness and loneliness, including anxious and depressive feelings related to the COVID-19 emergency. Since telecommunication in the medical field can be expected to improve the depression and well-being of children with NDDs, it is essential to build and improve the system in Japan.

### Limitations

The first of the study's limitations relates to the sample size of patients analyzed, which was small, even though the questionnaire collection rate was 100%. It was because there were a limited number of participants due to the single-center study. The second is that it is unclear whether the QOL of children was lower during the COVID-19 pandemic than before because the QOL of children with NDDs is usually significantly lower than that of the general child population (18, 36, 43). Third, the clinical characteristics of the children who participated in this study might differ from those throughout Japan because there were regional differences in infectious disease pandemics based on population density. Forth, it was impossible to accurately diagnose sleep disorders, as there were no interviews by doctors regarding children's sleep patterns and no sleep diaries from which to glean information. In the future, longitudinal studies analyzing sleep diaries of children with NDDs gain a clearer understanding of sleep disorders.

## Conclusion

Among the study sample, 46.5% of children with NDDs had changed sleep patterns during the COVID-19 stay-at-home period. Of these, 19 children (22.1%) also showed a high tendency for depression and anxiety, respectively. Changed sleep patterns were associated with decreased QOL and internalized symptoms. The decreased QOL of children with changed sleep patterns was predicted by high depression. In addition, low QOL in children with unchanged sleep patterns was predicted by high levels of depression and low current mood status.

Since the adjustment of sleep schedule was associated with depressive states improvement and also their QOL, it is recommended that clinicians focus on children's regular sleep schedule as manifested during the COVID-19 stay-at-home period. The QOL and psychiatric status had to be evaluated by self-report as much as possible. Furthermore, it is necessary for children's keeping regular sleep schedules to maintain a system for linkage of education, welfare services, and medical care even in critical situations.

## Data Availability Statement

The raw data supporting the conclusions of this article will be made available by the authors, without undue reservation.

## Ethics Statement

The studies involving human participants were reviewed and approved by the Institutional Review Board of the Shimada Ryoiku Center Hachioji (Shimahachi-2001). Written informed consent to participate in this study was provided by the participants' legal guardian/next of kin.

## Author Contributions

RU, TO, and HO decided on the conception and design of the study. YO, HI, MS, YKo, CKo, YN, KA, AI, NS, YM, CKa, MK, and HO jointly carried out the acquisition of data (participant collection and data curation). RU and YKi performed the data analysis. RU wrote the manuscript. TO supervised this work and assisted with the writing of the manuscript. All the authors have approved the final article.

## Funding

This work was supported partly by the Meiji Yasuda Mental Health Foundation, Japan (Grant Number 2020-1-007 to RU) and the Intramural Research Grant for Neurological and Psychiatric Disorders of NCNP, Japan (1–4 and 2–7 to TO).

## Conflict of Interest

The authors declare that the research was conducted in the absence of any commercial or financial relationships that could be construed as a potential conflict of interest.

## Publisher's Note

All claims expressed in this article are solely those of the authors and do not necessarily represent those of their affiliated organizations, or those of the publisher, the editors and the reviewers. Any product that may be evaluated in this article, or claim that may be made by its manufacturer, is not guaranteed or endorsed by the publisher.
